# Ecological traps: evidence of a fitness cost in a cavity-nesting bird

**DOI:** 10.1007/s00442-021-04969-w

**Published:** 2021-06-21

**Authors:** Ronalds Krams, Tatjana Krama, Guntis Brūmelis, Didzis Elferts, Linda Strode, Iluta Dauškane, Severi Luoto, Agnis Šmits, Indrikis A. Krams

**Affiliations:** 1 Department of Biotechnology, Daugavpils University, Daugavpils, 5401 Latvia; 2 Chair of Plant Health, Estonian University of Life Sciences, 51006 Tartu, Estonia; 3 Department of Botany and Ecology, Faculty of Biology, University of Latvia, Riga, 1004 Latvia; 4 English, Drama and Writing Studies, University of Auckland, Auckland, 1010 New Zealand; 5 School of Psychology, University of Auckland, Auckland, 1010 New Zealand; 6 Latvian State Forest Research Institute “Silava”, Salaspils, 2169 Latvia; 7 Department of Zoology and Animal Ecology, Faculty of Biology, University of Latvia, Riga, 1004 Latvia; 8 Institute of Ecology and Earth Science, University of Tartu, Vanemuise 46, 51014 Tartu, Estonia

**Keywords:** Ecological traps, Cavity-nesting birds, Great tits, Fitness cost, Resources, Behavioral ecology

## Abstract

Habitat quality has direct effects on the evolutionary fitness of breeding organisms, which is why it is believed that animals tend to have an evolved preference for the best possible habitats. However, some animals may mistakenly choose to reproduce in habitats that decrease their fitness, resulting in ‘ecological traps’. In this study, we tested whether great tits (*Parus major*) attracted to areas affected by outbreaks of the great web-spinning sawfly (*Acantholyda posticalis*) had fitness detriments characteristic of ecological traps. Sawfly larvae consume pine needles, which decreases resource availability for birds co-habiting the forest. Using artificial nesting sites, we found that great tits inhabiting areas of sawfly outbreaks had similar clutch sizes as tits breeding in healthy forest patches; however, the fledgling number was significantly lower, and fledgling condition was worse in the damaged forests. While moth larvae are the most important food for bird nestlings, the forest patches damaged by sawflies had lower larval biomass. Although most ecological traps occur in environments altered by humans, this study shows that pest insects can lower habitat quality, forming ecological traps. Our results indicate that attracting cavity-nesting birds should be done with caution because it may negatively impact birds’ nutritional status and reproductive fitness.

**Supplementary Information:**

The online version contains supplementary material available at 10.1007/s00442-021-04969-w.

## Introduction

Evolution creates variation in the genetic tapestry of life via natural selection. One of the principal drivers of natural selection is adaptation to different environments or ecological niches: some genetic variants become favored over others in certain environments (Schluter 2009; Luoto 2019a, b; Rees et al. 2020), resulting in variation in the diverse forms that life takes (Darwin 1859). Yet when environments change rapidly, and when organisms lack adequate genetic, behavioral, and/or phenotypic plasticity, organisms may end up choosing habitats that are detrimental to their fitness. Such outcomes are termed ‘ecological traps’ (Sherley et al. 2017; Sun et al. 2020). Ecological traps are becoming increasingly salient features in behavioral ecology because of human-induced environmental modifications (Hale and Swearer 2016). Ecological traps have three general criteria: (i) individuals prefer one habitat over another (a ‘severe’ trap) or equally prefer multiple habitats (an ‘equal preference’ trap); (ii) fitness (or a reasonable surrogate measure) differs between habitats; and (iii) fitness is lower when animals exploit the (equally) preferred habitat (Robertson and Hutto 2006; Hale and Swearer 2016). There are various ways by which ecological traps arise. Animals may mistakenly prefer habitats where their fitness is reduced because they have not experienced such conditions during their individual and evolutionary history (Hale and Swearer 2017). Animal survival and reproduction can also be impaired in habitats restored by humans if management activities result in an ecological trap (Hale and Swearer 2017).

Insects are crucial parts of forest ecosystems worldwide where they serve as food sources to other forest dwellers and perform the role of pollinators, omnivores, herbivores, carnivores, and decomposers. Insects often attack forest crops by decreasing timber resources. Several forest pest species experience population cycles in which populations remain low for several years and are followed by outbreaks (population explosions). Outbreaks of insects are considered to be major sources of habitat disturbance in forest ecosystems (Barbosa et al. 2012; Moulinier et al. 2013), altering vegetation characteristics (Dennison et al. 2010; Man and Rice 2010; Yang 2012; Karlsen et al. 2013), organismal interactions, and structure and density of consumer populations (Vindstad et al. 2015).

Pest insects and their outbreaks can be traditionally controlled by insecticides. However, agrochemicals often harm biological diversity, including all other beneficial arthropods, which substantially impairs ecosystem services provided by biodiversity (Daily and Matson 2008). Regulation of pests by attracting and enhancing natural enemies of insects is an alternative approach used in agriculture and forestry practice (Swinton et al. 2007; Tscharntke et al. 2012). Bird predation has an important role in biological control (Holmes et al. 1979; Langelier and Garton 1986; Duan et al. 2015) by reducing numbers of pest insects and significantly decreasing the frequency of outbreaks (Solomon et al. 1976; Torgersen et al. 1984). Birds have also been shown to reduce pest damage and substantially increase commercial fruit and coffee production (Mols and Visser 2002, 2007; Mols et al. 2005; Kellermann et al. 2008; Johnson et al. 2010; Jedlicka et al. 2011).

Biological control by provisioning nest boxes for insectivorous birds is a commonly used approach to attract hole-nesting birds, especially in Europe (Fischer and McClelland 1983; Gosler 1993; Kirk et al. 1996; Tilgar et al. 1999; Mols and Visser 2002, 2007; Mols et al. 2005; Mänd et al. 2005). The use of nest boxes has promoted biological research and led to significant progress in our understanding of ecological, physiological, and behavioral processes in birds including the impact of climate change on biodiversity (Lambrechts et al. 2010; Møller et al. 2014; Vaugoyeau et al. 2016; Samplonius et al. 2018). Putting up nest boxes is a simple method to encourage avian populations at the sites of insect outbreaks (Mols and Visser 2002; Jedlicka et al. 2011). Some birds, such as great tits (*Parus major*), show a striking preference for artificially made nest boxes over natural tree cavities (Drent 1984) because artificial nest boxes are constructed to minimize nest predation, humid microclimate, nest soaking, and improve nest illumination (Wesołowski 2011; Maziarz et al. 2016). Provisioning of nest boxes makes it easy to compensate for naturally low availability of cavities, which is a limiting factor especially in forest plantations. As cavities are among the most important cues for habitat selection of cavity-nesting birds (Hildén 1965), abundant nest boxes make an area attractive and thus the density of nesting bird can be raised well above naturally occurring densities. Birds can be attracted independent of the actual amount of resources available in the habitat (Mänd et al. 2005; Kilgas et al. 2007). This makes it possible to attract cavity-nesting birds to ecological traps or sink habitats that are preferred habitats where individual fitness does not increase or where mortality exceeds the birth rate (Gates and Gysel 1978; Delibes et al. 2001; Donovan and Lamberson 2001; Kokko and Sutherland 2001; Schlaepfer et al. 2002; Kristan 2003).

Interestingly, some bird species positively respond to the increased density of leaf-eating autumnal moth (*Epirrita autumnata*) larvae because they provide an unlimited food source for adult individuals and their offspring during outbreaks. This causes breeding nomadism in the brambling (*Fringilla montifringilla*) by attracting this passerine bird to birch forests affected by outbreaking *E. autumnata* (Mikkonen 1983; Hogstad 1985; Lindström 1987). However, some other birds do not react at all or respond negatively to outbreaks by *E. autumnata* (Enemar et al. 2004). For example, the redpoll (*Carduelis flammea*) is to some extent dependent on birch seeds, a food supply that is not available in subalpine birch forest affected by *E. autumnata* (Enemar and Nyström 1981). This suggests that outbreaking insects deteriorate the environment even though their larvae constitute a considerable part of the food of local birds (Hogstad 1988). The grazing larvae, for instance, affect the vegetation in the form of defoliation, reduced flowering, and seed production which may have a negative effect on other arthropods, lowering the overall quality of the environment. Finally, this reduces the number of outbreaking insects themselves and forces most of the birds to leave the area (Selås et al. 2001; Enemar et al. 2004).

Putting up nest boxes in the forest patches affected by insect outbreaks may attract cavity-nesting birds to ecological traps. In this study, we tested whether great tits breeding in pine forests heavily damaged by outbreaking of the great web-spinning sawfly (*Acantholyda posticalis*) suffer fitness costs. The great tit is a common bird species in Latvia and readily accepts nest boxes to breed in any kind of forest and parkland. Within the breeding season, great tits mainly forage for insect larvae, which is the preferred food for their nestlings and fledglings (Rytkönen and Krams 2003). We provided nest boxes in mature Scots pine (*Pinus sylvestris*) forest stands both affected (loss of foliage) and non-affected by sawflies. Natural cavities were hardly available in either environment. Hole-nesting birds have been traditionally considered as predators that can affect defoliator pest outbreaks, which is why foresters traditionally put up nest boxes in forest stands affected by the sawfly and moth pest species (Bičevskis 2005; Jankevica 2008; Šmits 2005).

Great web-spinning sawfly adults emerge from the soil and females lay eggs on needles of Scots pine in June (Voolma et al. 2016). Sawfly larvae consume the needles of pines and feed on the needle substrate until the beginning of August. At the fourth larval instar stage the larvae move to the soil where they stay for two to five years before they emerge after a short pupation (Ghimire et al. 2013). The highly variable larval stage makes outbreaks of great web-spinning sawflies unpredictable (Ghimire et al. 2013). Importantly, patches damaged by a web-spinning sawfly outbreak are easy to distinguish from healthy patches because in the damaged areas pines are strikingly defoliated.

While we predicted similar clutch sizes between the patches damaged by web-spinning sawflies and healthy patches, we expected smaller fledgling numbers, lower fledgling body mass, and shorter tarsus lengths due to malnutrition in the nest boxes located in the patches damaged by sawflies. We also studied larval biomass in patches occupied by great tits to estimate food resources available to their nestlings. As larval biomass can be expected to be related to the amount of available foliage, we used estimates of live tree crown volume and canopy cover as indirect measures of larval biomass (Brūmelis et al. 2020).

## Materials and methods

### Study area, nest boxes, and birds

The breeding ecology of great tits was monitored near Daugavpils, southeastern Latvia (55.55° N, 26.34° E). The study area covers Scots pine stands affected by an on-going mass outbreak of the great web-spinning sawfly. The outbreak was first observed in summer 2013. This is a second observed outbreak of this pest in Latvia. The previous outbreak was observed some 40 km eastwards during 1966–1982. Prolonged outbreaks are typical for great web-spinning sawflies. Years of intensive flight are followed by years when the majority of larvae fall in diapause. Consequently, years with heavy tree defoliation are followed by years when trees are able to partly recover their foliage. This study was conducted in 2019 when flight activity was low and the larvae of great web-spinning sawflies were hardly available as a food resource for birds in the spring–summer period.

**Fig. 1:**
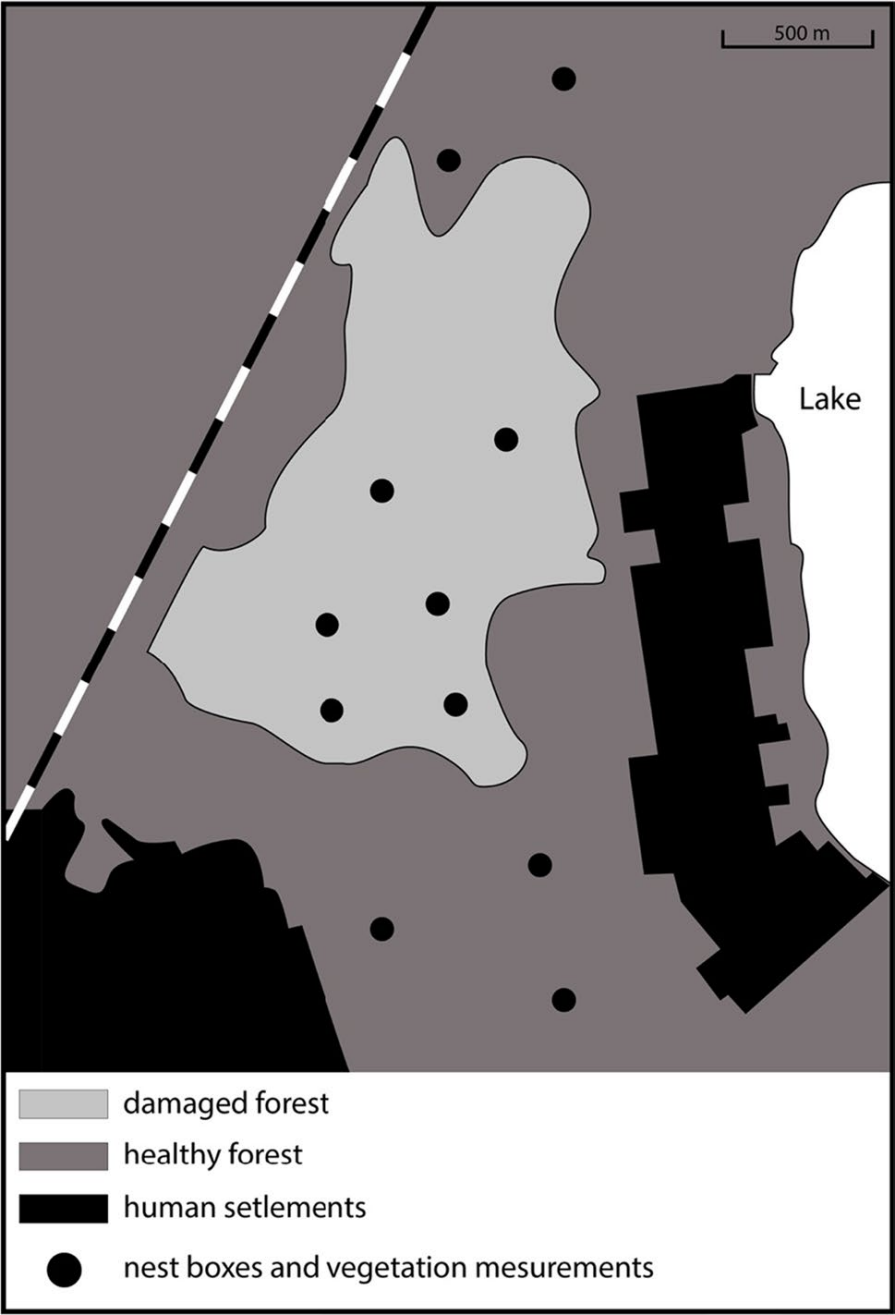
The study sites in a pine forest in the surroundings of Daugavpils. Filled circles denote the sites where pine condition was studied and where the nest boxes were located

Nest boxes were mounted on pine trunks at a height of about 3.0 m. The internal size of the nest boxes was 0.13 × 0.13 × 0.25 m, and the diameter of the entrance was 0.036 m. Breeding success, fledgling number, their body mass, and tarsus length were recorded in two contrasting types of forest patches—the pine forest damaged by web-spinning sawflies and a nearby healthy pine forest. We chose six areas in the affected pine forest and five areas in the nearby healthy forest (Fig. 1). We put up 12 nest boxes in each of these patches (72 nest boxes in the affected forest and 60 nest boxes in the healthy forest). Out of 132 nest boxes, great tits occupied 34 nest boxes in the damaged forest and 31 nest boxes in the healthy forest (65 nest boxes in total). Great tit offspring successfully fledged in 59 nest boxes (30 nest boxes in the damaged areas and 29 nest boxes in the healthy areas). The total area of the damaged forest was c. 120 ha. The total size of studied patches with nest boxes was c. 3.8 ha. The distance between study patches (each containing 12 nest boxes) was at least 480 m. To avoid competition (Dhondt 2011), the distance between neighboring boxes was c. 50 m in each of the 11 study patches.

The nest boxes were checked regularly to record basic breeding parameters, such as the number of eggs and the number of fledglings, which is an indication of breeding success. To assess offspring quality, all nestlings were weighed with a Pesola spring balance to a precision of 0.1 g, and their tarsi were measured with sliding calipers to the nearest 0.1 mm on day 15 posthatch (Kilgas et al. 2006).

### Food resources

We estimated the amount of food resources available in each forest patch by using the frassfall method (Rytkönen and Krams 2003). In brief, frass production by larvae was measured using plastic funnels (diameter 35 cm) with a paper coffee filter (size 1 × 4) attached to each funnel. The filter lets rainwater go through but frass produced by herbivory larvae is retained inside the filter. We used three funnels in each study patch (*n* = 33). The funnels were attached to trunks of randomly chosen pines, and the distance between the funnels was c. 60 m. As soon as the first nestlings in the patch reached the age of 7 days, the funnels were placed for a period of 4 days. The filters with the frass were preserved in a freezer. The frass production was determined by counting the frass items in each filter, and the average diameter of the frass items was determined by measuring randomly sampled frass items in each filter with an ocular micrometer. We estimated larval biomass from frass dry mass by using an allometric relationship between frass diameter and frass dry mass (Rytkönen and Orell 2001) and the equation by Tinbergen and Dietz (1994). As we could not discriminate between frass produced by larvae of moths and sawflies (Zandt 1994), this part of the research provided an estimation of the total food resources available in each forest patch.

### Tree canopy in the patches of sawfly outbreak and in the unaffected patches

We studied how the condition of forest patches affects breeding parameters of great tits. We distinguished between healthy (< 25% foliage loss), damaged (25–75% foliage loss), and dead trees (< 25% foliage remaining) (Brūmelis et al. 2020). We measured the following three condition parameters: (1) total canopy cover of pines (%), (2) the relative number of dead and dying trees with 75–100% loss of needles due to web-spinning sawfly damage (%), and (3) the total tree crown volume (m^3^ ha^−1^). In a recent study on allometric relationships between tree crown parameters, we proposed that, while tree crown parameters are usually ignored in studies on food resources due to difficulty of measurement, there are simple-to-measure parameters that could be used in studies to estimate food resources of animals (Brūmelis et al. 2020). The canopy cover is the layer formed by the branches and leaves of trees. The cover has higher values when it is continuous and much smaller when it is discontinuous. The relative number (%) of dead trees reflects the rate of damage done by a pest. High amounts of leaf (needle) damage eventually leads to the death of a tree. The total volume of tree crowns in the patch is important because it reflects the total amount of substrate that insectivorous birds can use to collect their food.

Four circular plots sized 10 m^2^ were set up in azimuth directions at a 50-meter distance from a central location in each patch. Canopy cover was estimated with a gridded concave mirror (Forest densitometer) in each plot in four azimuth directions at a central point offset at least by a 2-meter distance from the nearest tree. Briefly, the grid on the mirror is used to count points at crossing lines that coincide with the tree canopy on the mirror, calculated as percent canopy cover (Brūmelis et al. 2020).

In each plot, diameter (DBH) of all trees at a height of 1.3 m was measured. In addition, measurement of tree crown parameters (height to top and base of the live tree crown, and width of the tree crown in two perpendicular directions) were made in each plot for 2–4 trees with different size and extent of damage. A Haglof VL5 vertex was used to measure height to top and base (lowest living branch) of the live tree crown. A GRS densitometer was used to precisely locate edge of the crown for width measurements. Tree crown measurements were made for 76 healthy and 16 damaged (more than 25% of needles lost) pine trees. Tree crown volume was estimated as an ellipsoid, as suggested for practical purposes for Scots pine (Rautiainen et al. 2008). The allometric relationship between stem diameter and crown volume for sampled trees for crown parameters was used to estimate volume for all trees in plots using an exponential regression model, separately for healthy [volume = 10.529588*EXP(0.068715*DBH)] and damaged [volume = 3.85498*EXP(0.09189*DBH)] trees. The exponential model was found to best explain the relationship between DBH and crown volume (R^2﻿^ = 0.525 and R^2^ = 0.605 for healthy and damaged trees, respectively), and was superior or similar to a linear and power relationship, respectively. For the calculations, we also included data from 82 pine trees measured in this study area (Brūmelis et al. 2020). The total tree crown volume per hectare in the stands was then estimated.

### Data analyses

We used a Bayesian linear mixed-effects models (LMER) and generalized linear mixed-effects models (GLMM) as implemented in the R 4.0.2. (R Core Team 2020) library brms (Bürkner 2017) to analyze the effects of stand parameters (independent variable) on the bird parameters (dependent variable). Separate models with one fixed factor and one dependent variable were implemented for each combination of stand parameters: total canopy cover, rate of dead trees, total canopy volume; and bird parameters: clutch size (Poisson GLMM), proportion of fledglings (binary logistic GLMM), body mass (LMER), tarsus length (LMER). In all models plot ID was set as a random factor to account for pseudoreplication. For models with body mass and tarsus length, nest ID was added as a nested random factor within plot ID. The number of iterations was set to 2500 for each of four chains. Rhat values (all close to ~ 1.00) were used to assess the convergence of the models. *P* values for the models were calculated with R library bayestestR (Makowski et al. 2019) function p_map. Spearman correlation analysis was used to assess relationships between stand parameters (canopy volume, total pine canopy cover, the proportion of dead trees) and larval biomass.

## Results

### Larval biomass in damaged and healthy forest patches

**Fig. 2:**
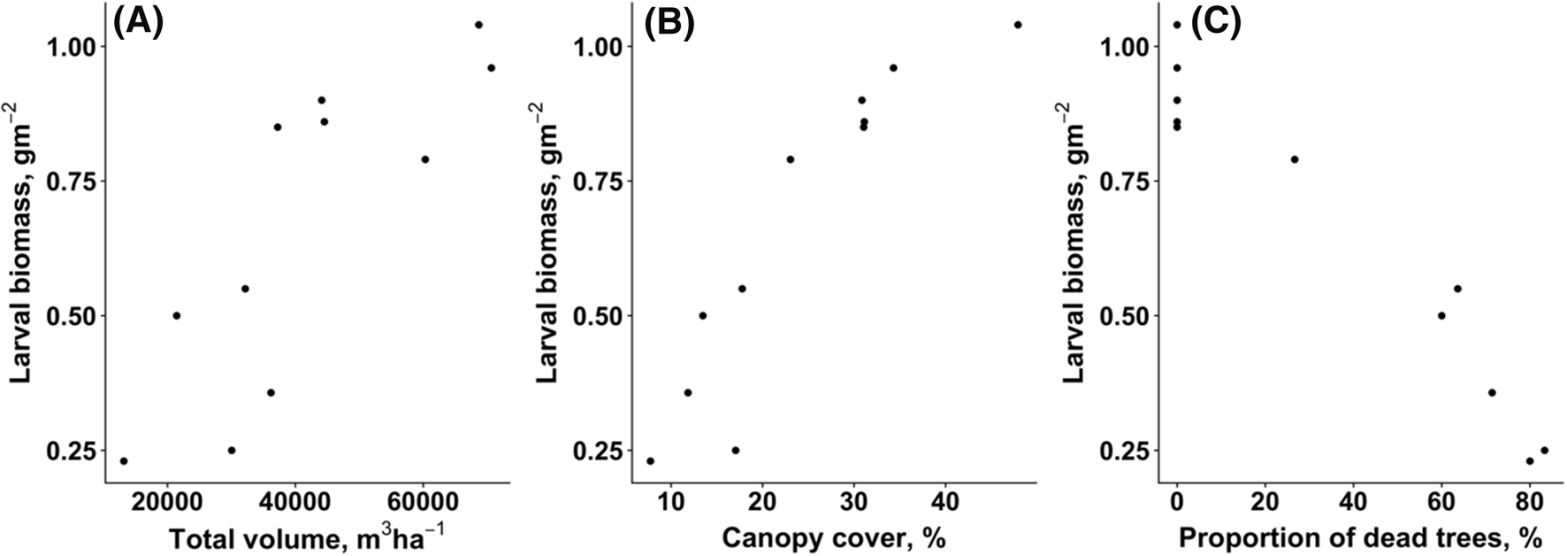
Correlations between larval biomass and total canopy volume (**A**), total pine canopy cover (**B**), and rate of dead trees (**C**)

The overall biomass of canopy-dwelling insect larvae during the nestling period of great tits was significantly associated with sawfly damage. Larval biomass in the canopy increased in patches with greater canopy volume (*r*_*s*_ = 0.882, *P* = 0.001; Fig. 2A), increased in patches with greater total pine canopy cover (*r*_*s*_ = 0.945, *P* < 0.001; Fig. 2B), and decreased in patches with a high number of dead trees (*r*_*s*_ = − 0.934, *P* < 0.001; Fig. 2C).

### Clutch size

**Fig. 3:**
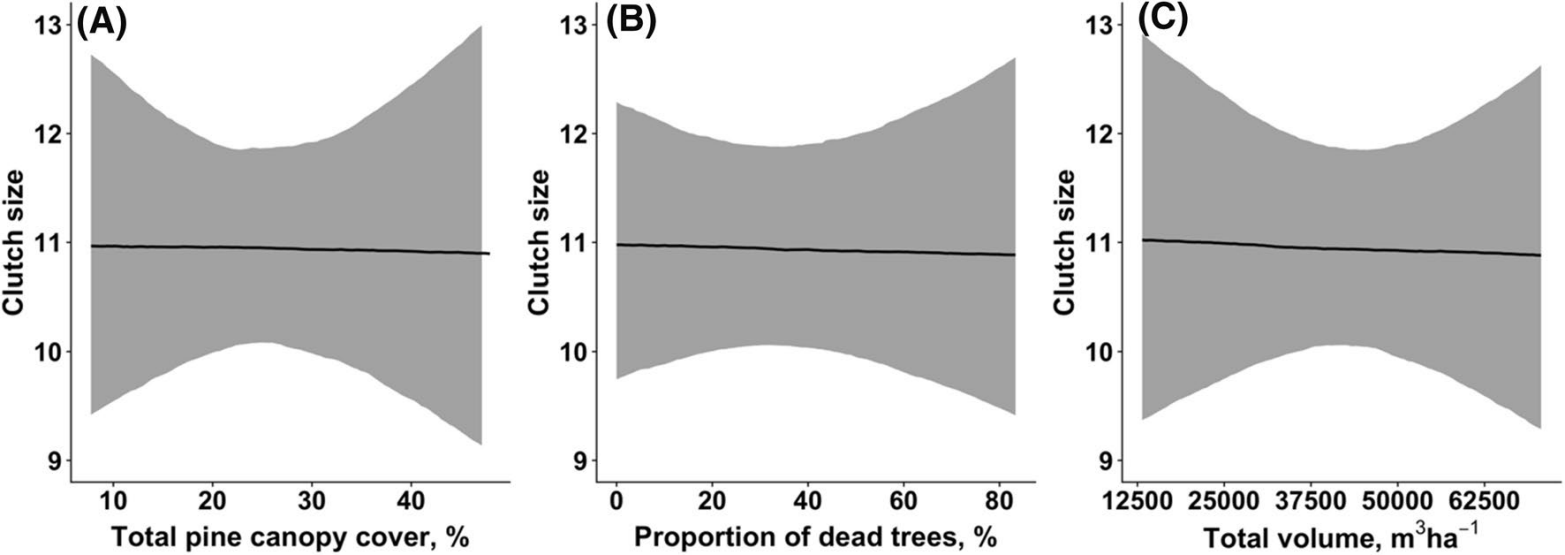
Associations between the clutch size of great tits and total pine canopy cover (**A**), rate of dead trees (**B**), total canopy volume (**C**). Solid lines show the estimated trendlines by the model, and grey-shaded areas represent 95% credibility intervals

Sawfly damage was not significantly associated with clutch size in great tits. Clutch size did not depend on the total pine canopy cover [Slope estimate: − 0.000, Credibility interval (CI): (− 0.007, 0.007), *P* = 1.00, Fig. 3A], the proportion of dead trees [Estimate − 0.000, CI (− 0.002, 0.002), *P* = 0.984, Fig. 3B], nor on total canopy volume [Estimate − 0.004, CI (− 0.087, 0.078), *P* = 0.992, Fig. 3C]. We did not observe second clutches of great tits in the forest damaged by the outbreak of great web-spinning sawflies, while 58.6% (*n* = 17) of the great tits had second clutches in the forest unaffected by the pest.

### Number of fledglings

**Fig. 4:**
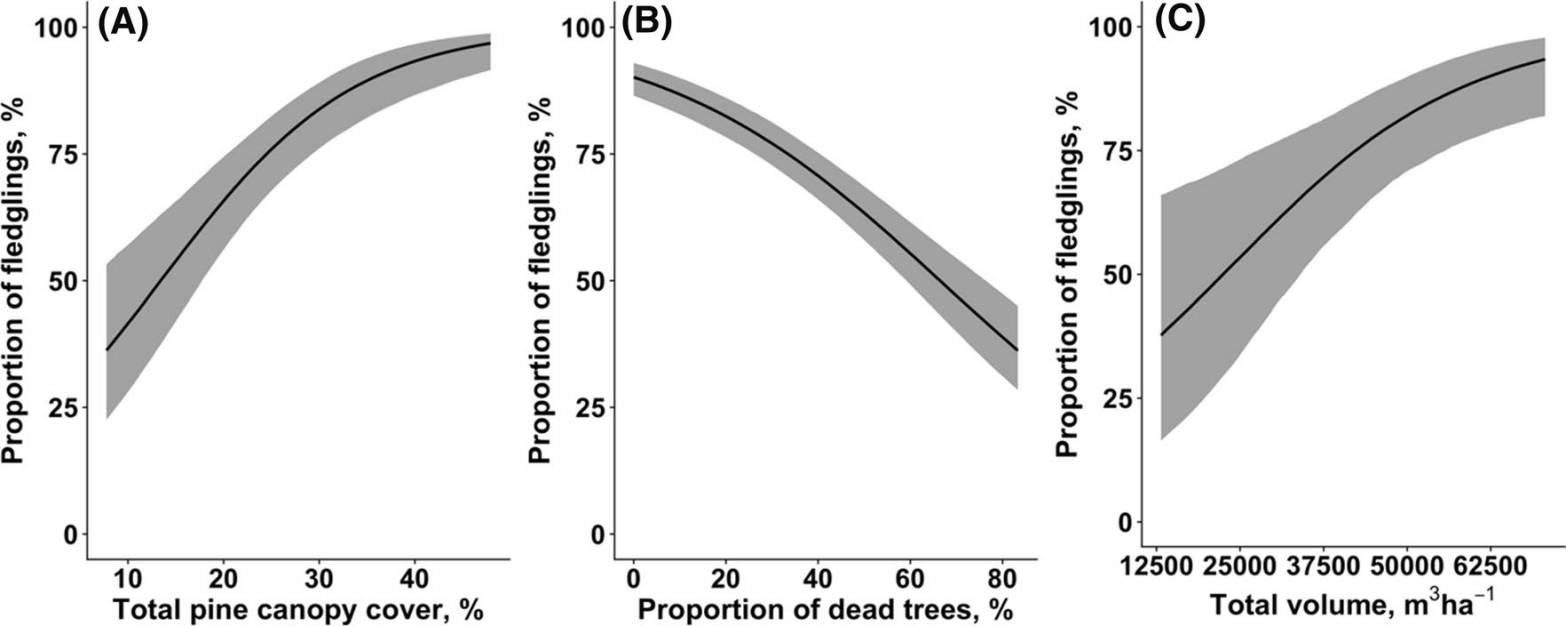
Associations between the proportion of fledglings per clutch and total pine canopy cover (**A**), rate of dead trees (**B**), and total canopy volume (**C**). Solid lines show the estimated trendlines by the model, and grey-shaded areas represent 95% credibility intervals

The rate of sawfly damage reduced the proportion of the young fledged per clutch. The proportion of fledglings per clutch increased with the total pine canopy cover [Estimate 0.099, CI (0.062, 0.135), *P* < 0.001, Fig. 4A], decreased with the number of dead trees [Estimate − 0.033, CI (− 0.040, − 0.027), *P* < 0.001, Fig. 4B], and increased with total canopy volume [Estimate 0.973, CI (0.371, 1.565), *P* = 0.016, Fig. 4C].

### Fledgling body mass

**Fig. 5:**
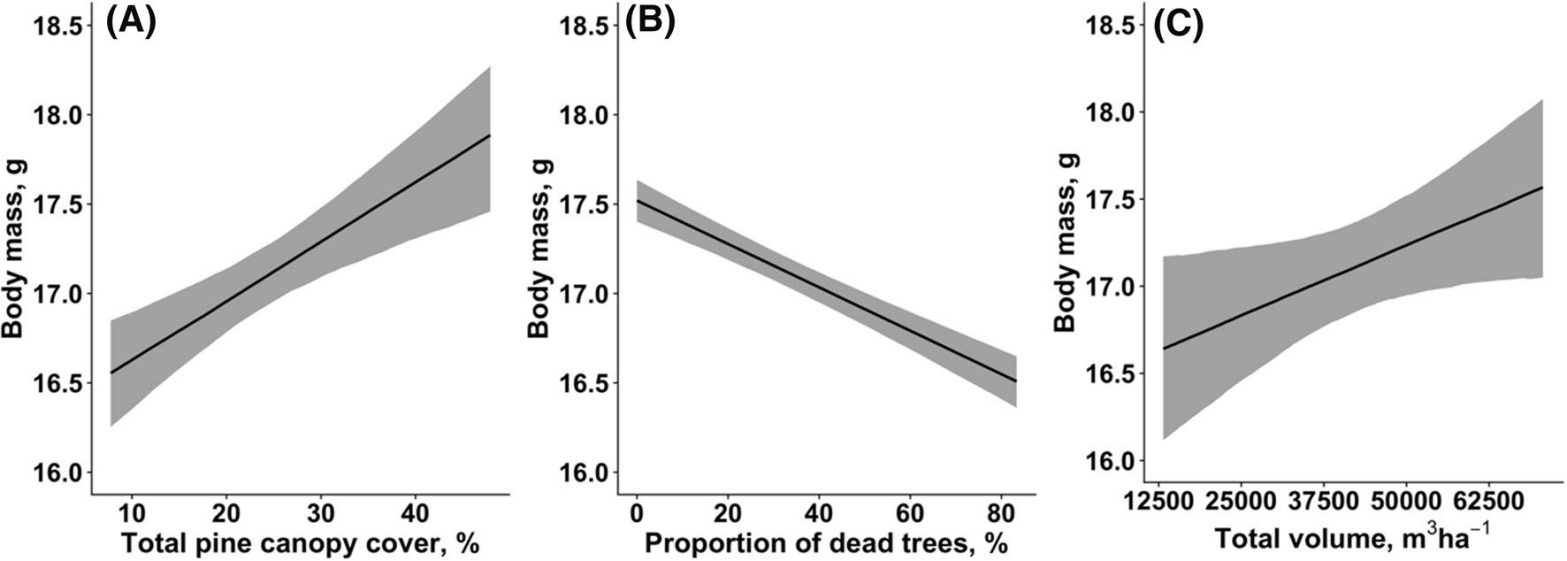
Associations between fledgling body mass and total pine canopy cover (**A**), rate of dead trees (**B**), and total canopy volume (**C**). Solid lines show the estimated trendlines by the model, and grey-shaded areas represent 95% credibility intervals

The extent of damage caused by the sawfly outbreak was significantly associated with fledgling body mass in great tits. We found that body mass of fledglings increased with the total pine canopy cover [Estimate 0.033, CI (0.017, 0.048), *P* < 0.001, Fig. 5A], declined with increasing number of dead trees [Estimate − 0.012, CI (− 0.014, − 0.010), *P* < 0.001, Fig. 5B], and increased with the total canopy volume [Estimate 0.289, CI (0.006, 0.567), *P* = 0.084, Fig. 5C].

### Fledgling tarsus length

**Fig. 6:**
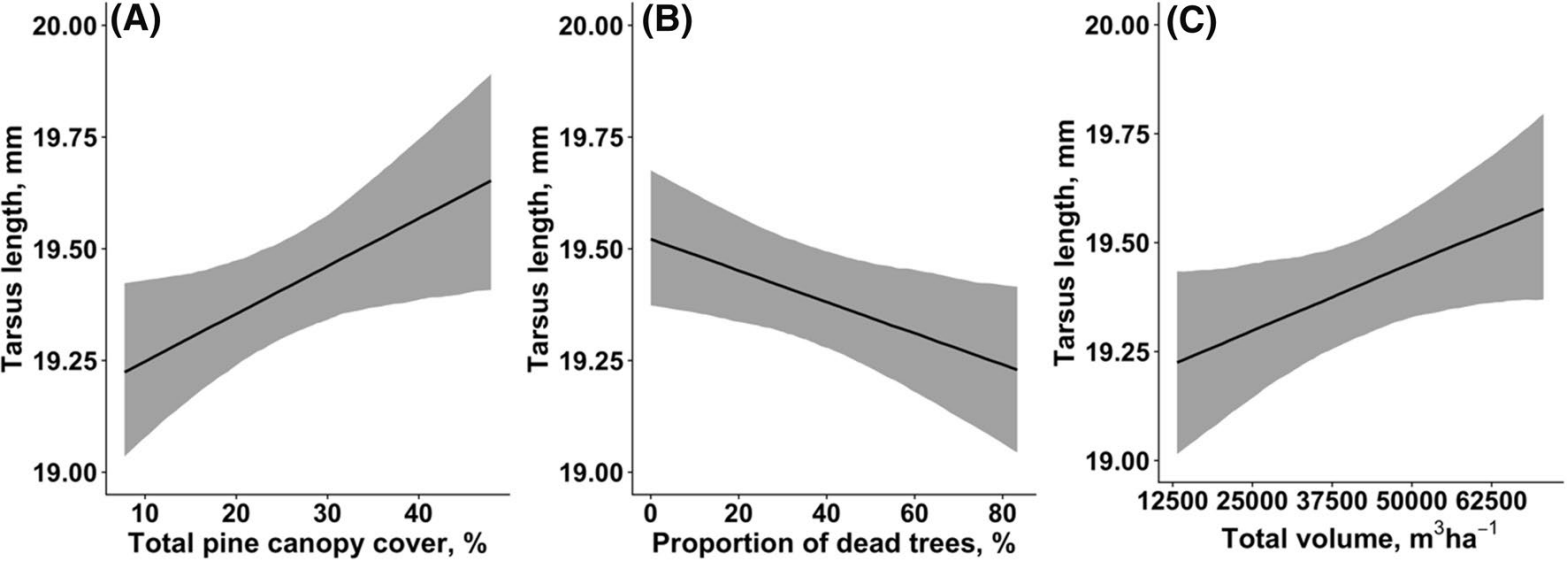
Associations between fledgling tarsus length and total pine canopy cover (**A**), rate of dead trees (**B**), and total canopy volume (**C**). Solid lines show the estimated trendlines by the model, and grey-shaded areas represent 95% credibility intervals

Great web-spinning sawfly outbreak was significantly associated with fledgling tarsus length. We found that tarsus length of fledglings increased with the total pine canopy cover [Estimate 0.011, CI (0.001, 0.020), *P* = 0.071, Fig. 6A], declined with increased number of dead trees [Estimate − 0.004, CI (− 0.007, − 0.001), *P* = 0.061, Fig. 6B], and increased with total canopy volume [Estimate 0.110, CI (0.000, 0.222), *P* = 0.128, Fig. 6C].

## Discussion

Based on the canopy indices like tree crown volume and proportion of dead trees, the patches represented a gradient from severely damaged to healthy stands. Further, the strong correlations of larval biomass with tree canopy volume and canopy cover suggest that in similar studies, the easy-to-measure canopy parameters might be used a proxy for the amount of food resources. We found that clutch size of great tits did not statistically correlate with any of the canopy indices (used as an indirect representative of the outbreak of great web-spinning sawflies). This suggests that birds chose their breeding habitats based on the availability of cavities suitable for breeding. However, the number of fledglings was lower, and their condition was substantially poorer in the forest damaged by the sawfly outbreak. Larval biomass was significantly greater in the healthy forest area characterized by greater total canopy cover and total canopy volume and lower rate of dead trees than in the damaged forest. *A. posticalis* larvae develop later in the season when young birds have already fledged their nests and did not serve as a food source. Overall, our results indicate that the damaged forest area constitutes an ecological trap for the birds that attempted to breed in this type of forest.

‘Severe ecological traps’ occur when animals prefer to occupy poor-quality habitats over habitats of good quality. Ecological traps can generally arise when the behavior and preferences of the organism do not match its environment—a mismatch caused by serious changes in the environment of the organism while its behavior remains the same as before the environmental changes (Kokko and Sutherland 2001; Schlaepfer et al. 2002; Mänd et al. 2005; Hale and Swearer 2016). In this study, we deal with an ecological trap that meets all three criteria suggested by Robertson and Hutto (2006) and Hale and Swearer (2016). Cavities as the main limiting resource for hole-nesting birds can be completely absent in managed pine plantations where nest boxes are put up to compensate for the lack of natural cavities. It is important to note that habitat quality of wild organisms can be impaired, and habitats can be transformed in low-quality patches or even ecological traps caused not only by humans (Demeyrier et al. 2016). We show that forests damaged by pest insects are transformed into ecological traps in such cases when artificial nest boxes are provided for hole-nesting birds. While great tits are instrumental in fighting sawflies in the areas of their outbreaks, the attraction of birds to these forest patches leads to maladaptive outcomes and significantly decreases fitness parameters of the birds.

The results of this study suggest that the poor availability of insect larvae in the outbreak area makes food abundance a crucial factor in decreasing fitness parameters in breeding great tits. Great web-spinning sawfly causes substantial damage to pine canopies by eating their needles. In the great tit, insect larvae form up to 73% of the nestling diet (Rytkönen and Orell 2001) and this species is highly dependent on herbivorous insects and their larvae during the nestling period. However, outbreaking sawflies destroy most of the branches, weaken pines, and even kill individual trees, thereby making foraging substrate less available for next generations of sawflies and other herbivorous insects. The ability of adult great tits to compensate for low habitat quality is limited. This is because great tits primarily search for larvae and do not totally switch to some other, more abundant food during the nestling period (Robinson and Holmes 1982; Holmes and Schultz 1988). Another reason for the inability to compensate for low habitat quality is that the birds typically collect food for their nestlings within 50–70 m from the nest (Rytkönen and Orell 2001; Rytkönen and Krams 2003). The inability of parents to bring enough food, together with the low leaf eating larvae biomass in the patches affected by web-spinning sawflies, causes malnutrition of nestlings. They grow more slowly and are smaller at the time of fledging in the outbreak areas. Malnutrition and probably also competition between nestlings cause higher nestling mortality which results in a significantly lower number of fledglings in the outbreak area compared to the healthy forest.

One of the shortcomings of this study was that we could not estimate the fledgling survival and recruitment rate of great tits breeding in the damaged and healthy forest patches. Our results show that adult great tits have never attempted to have second clutches in the damaged forest. They left this area together with their fledglings as soon as their offspring fledged (*pers. obs.*). We did not continue this experimental study for one more year, which precluded us from estimating the recruitment rate in the outbreak area to compare this with recruitment rates in the healthy forest. We highly recommend that other researchers continue their studies for at least two breeding seasons of their study subjects to better understand the effects of ecological traps.

The results of this study may have important conservation and management implications. First, although hole-nesting birds are easy to attract to particular areas where they can be used as biological control agents to fight agricultural and forestry pests, it is important to discuss the extent to which it is ethical to lure birds to ecological traps. It is equally important to develop the theory of ecological traps because of our limited ability to predict the formation of ecological traps, identify them when they do exist, and to mitigate their impact (Hale and Swearer 2016; Robertson and Hutto 2006). However, we show that forest patches deteriorated by pest insects are easy to identify, which may help to prevent the attraction of insectivorous birds to the area of the ecological trap. Our results also raise the question of whether other human activities have the potential to turn large forest areas into ecological traps. For example, if modern forestry measures such as regular removal of understory trees and bushes from the plantations of coniferous forests reduces biomass of insects and simultaneously erecting nest boxes for insectivorous birds increases the density of birds above naturally occurring levels, it might form ecological traps at the level of populations, environmental niches, and ecosystems (Lindenmayer et al. 2008; Hale et al. 2015; Krama et al. 2015; Hale and Swearer 2016).

Finally, our results highlight the need to balance conservation efforts with research on habitat quality and the carrying capacity of ecosystems. For example, ecological traps may ruin an investment in the conservation of a species if the area contains too many competitors or its future quality is compromised. Ecological traps such as low-quality forests may also decrease landscape connectivity even if these traps result in minor immediate fitness consequences (Sánchez-Mercado et al. 2014; Hale et al. 2015). Low genetic heterogeneity of organisms in these areas can further decrease their fitness and reduce the success of conservation measures (Prunier et al. 2017).

## Conclusions

The use of nest boxes to attract cavity-nesting birds to areas of insect outbreaks is a traditional measure to protect forest ecosystems. However, these forests can become deteriorated by pest insects so that the attracted birds lack sufficient resources to feed their offspring. The results of this study show that ecological traps can arise in forest areas where humans attract insectivorous cavity-nesting birds to fight outbreaking insects. Cavities are the main limiting resource for birds nesting in nest boxes. By installing nest boxes, the density of birds can be easily raised above naturally occurring densities, thus exceeding the carrying capacity of bird habitats. We found malnutrition and higher mortality of offspring in the forest area affected by insect outbreaks. Our results suggest that the use of cavity-nesting birds in the biological control of insect pests should be done with caution because it may negatively impact birds’ reproductive fitness in areas of unintended ecological traps.

## Supplementary Information

Below is the link to the electronic supplementary material.

Supplementary file1 (XLSX 147 kb)
